# Dietary Adequacy in Older Adult Nursing Home Residents of the Northern Iberian Peninsula

**DOI:** 10.3390/nu16060798

**Published:** 2024-03-11

**Authors:** Nicolás Pidrafita-Páez, Joana Silveira, Elisabete Pinto, Luis Franco, Mª Ángeles Romero-Rodríguez, Mª Lourdes Vázquez-Odériz, NUTRIAGE Study Group

**Affiliations:** 1 Areas of Nutrition and Food Science and Food Technology, Department of Analytical Chemistry and Food Science, Faculty of Science, University of Santiago de Compostela, 27002 Lugo, Spain; nicolas.piedrafita@rai.usc.es (N.P.-P.); lourdes.vazquez@usc.es (M.L.V.-O.); 2 CBQF—Centro de Biotecnologia e Química Fina—Laboratório, Universidade Católica Portuguesa, Associado Escola Superior de Biotecnologia, Rua Diogo Botelho 1327, 4169-005 Porto, Portugal; joanaifsilveira@gmail.com (J.S.); ecbpinto@ucp.pt (E.P.); 3 EPI Unit—Institute of Public Health, University of Porto, Rua das Taipas, n° 135, 4050-600 Porto, Portugal; 4 Economic Analysis and Modeling Group, Instituto de Estudios y Desarrollo de Galicia (IDEGA), 15701 Santiago de Compostela, Spain; luis.franco@usc.es

**Keywords:** nursing homes, older adults, dietary intake, nutritional adequacy

## Abstract

According to the latest data, Spain (19.4%) and Portugal (21.8%) are the two European countries with the highest percentage of older adults. Concerns about diet quality are increasing, especially among institutionalized older people, who are at the greatest risk of malnutrition. To assess the adequacy of dietary intake of 186 older adults (>65 y) institutionalized in public nursing homes (NH) in Galicia (Northwest Spain) and the Porto district (Northern Portugal), a cross-sectional study has been carried out. The double-weighing method and a country-specific food composition database were employed for nutritional assessment. Nutritional adequacy was assessed based on the recommendations of the EFSA and WHO. Caloric intake in Spanish NHs was higher than in Portuguese facilities; 20.3% and 35.8% of Spanish and Portuguese residents, respectively, had a protein intake below the EFSA recommendation, and 46.2% and 44.9% of residents in Portugal and Spain, respectively, had lipid intakes below the reference intake range. Further, 45.5% of the residents in Portugal and 66.9% in Spain had a carbohydrate intake within the recommended range. Salt intake was higher than the recommendation for 67.0% and 32.3% of the residents in Spain and Portugal, respectively. This study supports the notion that institutionalized older adults are prone to significant nutritional inadequacies.

## 1. Introduction

Ageing can be accompanied by different diseases and deficiencies that can directly influence the nutritional needs of older people. In addition, these people more prone to nutritional deficits [1]. According to the latest data [2], Spain (19.4%) is slightly below the European Union (EU) average in terms of population ageing (20.3%), while Italy (22.8%), Greece (22.0%) and Portugal (21.8%) are the countries that have the highest percentage of older adults. In 2021, the northern region of Portugal, where 22.6% of people were over 65 years of age, was the fourth oldest area [3]. In Spain, the autonomous region of Galicia, where 25.2% of the population are over 65, ranked third for oldest population. In developed countries, this is a common phenomenon, and concerns about dietary quality are growing. Institutionalized older people are the ones with the highest risk of malnutrition or nutritional deficits. Diet is influenced by several factors present in nursing homes. These factors include the inadequacy of the menus offered in nursing homes, which can increase the risk of frailty, cardiovascular disease, osteoporosis, cachexia, malnutrition, and cognitive impairment [4]. In fact, even if nutrient and energy intake levels are satisfactory, their nutritional status can be affected by alterations in metabolic processes, drug–food interactions or altered requirements for certain nutrients [5]. The health costs of disease-related malnutrition in the older population are considerable, and are expected to increase further as the population ages [6]. The prevalence of malnutrition in Europe is 1–15% in non-institutionalized older adults, 25–60% in older adults in nursing homes, and 35–65% in hospitalized older adults [7]. Although malnutrition is a prognostic factor associated with morbidity, mortality and costs of care, nutritional problems in older adults are often overlooked or not addressed [8]. This suggests that the condition of older adults at risk of malnutrition should be investigated and improved. Apart from appetite, depression and other conditions, the dietary intake of older adults is largely determined by the quality of menus offered. Therefore, attention to the need for mealtime care is essential. Research conducted across various regions has illuminated the challenges and discrepancies inherent in nutritional care practices within residential care facilities for older adults. Studies conducted in the United States have underscored prevalent issues of malnutrition and inadequate food intake among residents, linking these concerns with adverse health outcomes and heightened mortality rates [9,10]. Similarly, research in Europe has identified substantial variations in nutritional care practices among different countries, highlighting the need to establish standardized guidelines and procedures to ensure optimal nutrition for institutionalized individuals [11]. Consequently, nutrition services amenable to implementation and support, aimed at alleviating malnutrition or improving long-term nutritional status, play a pivotal role in the nutritional care of residents. Food services that can be implemented and supported to reduce malnutrition or improve nutrition in the long term play a key role in the nutritional care of residents. Menus should be carefully planned and reviewed by a registered dietitian to ensure that they are healthy and meet nutritional, social, and cultural needs [12]. Several methods of dietary assessment have been used to monitor dietary intake in institutionalized older people, but the technique of accurately weighing food intake has been established as the gold standard [13]. However, it is uncommon to find studies that employ this technique due to the time that must be invested in each measurement. Using this technique, it will be possible to supply a more precise estimate of the nutritional adequacy of the menus served in nursing homes (NH). This study is necessary to ascertain the adequacy of the dietary intake of older adults in NH to intervene and improve this adequacy. Important factors that affect dietary intake, such as the quality of the menus and the size of the portions offered, could be optimized to comply with the recommendations for people over 65 years of age, developed by the European Food Safety Agency (EFSA) [14] and the World Health Organization (WHO) [15]. Furthermore, it is important to study whether differences emerge in comparisons between genders or even between countries.

## 2. Materials and Methods

### 2.1. Study Sample

This was a cross-sectional study. The dietary intakes of 186 residents (age > 65 y) were assessed; 118 were from NH in Spain (Galicia, Northwest Spain) and 68 were from Portugal (Porto district, Northern Portugal). In total, 7 NH (4 in Spain and 3 in Portugal) were analyzed. The NH were in major metropolitan areas. The care facilities in Galicia are under public management and those in Portugal are partially public-funded and partially private-funded. All the care facilities, except one in Portugal, have their own kitchen. Breakfast, lunch, and dinner are provided in all the care facilities, and, in addition, mid-morning and afternoon meals and supper are served in Portugal. The participants ate all their meals in the NH on the days of observation, and the older adults involved in this study ate meals without any texture-modified foods. Given that the weighing of the dishes served does not require the cooperation of the user, it is important to stress that this study is part of a larger research project in which self-reported information was included. This study is part of the NUTRIAGE research project (that seeks to find advanced solutions for healthy ageing through nutrition in the framework of the Galicia–North Portugal Euroregion) (ref.: 0659_NUTRIAGE_1_E). Exclusion criteria included the existence of severe end-stage organ failure, severe multisystemic organ failure, neoplasia in active treatment, expected survival of less than one month, inability to answer the questionnaires by the patient assessed according to the “Mini Mental State Examination” [16], diagnosis of psychiatric illness with eating disorders (anorexia, bulimia), acquired brain damage and/or previous history of alcoholism. The study was reviewed and approved by the Central Ethics and Research Committee of Galicia Autonomous Community (2017/542) and by the Ethics Commission of the Santa Casa da Misericórdia de Porto (Nutriage5set2017-ata 34).

### 2.2. Data Collection

Sociodemographic, clinical, and anthropometric data were collected by the NUTRIAGE project team, mentioned above, and by the medical staff of the institutions using validated questionnaires. Height was obtained using a calibrated stadiometer (Seca^®^ 206) with 0.1 cm accuracy. When this was not possible, because the participants could not stand up, height was estimated from a standard formula based on knee height [17] measured with a measuring tape (Seca^®^ 201,Hamburg, Germany) with 0.1 cm accuracy. Body weight (in kg) was measured with a portable mechanical scale (Seca^®^ 761, Hamburg, Germany) with 1 kg accuracy. When the older people could not stand up, weight was measured using a chair scale (Seca^®^ 954, Hamburg, Germany). Subsequently, Lipchitz’s [18] age-specific Body Mass Index (BMI) classification was considered (underweight: <22.0 kg/m^2^; normal weight: 22.0 ≤ BMI ≤ 27.0 kg/m^2^; excess weight: >27.0 kg/m^2^). Data were collected between October 2019 and March 2020.

### 2.3. Nutritional Intake

The analysis of nutritional intake was conducted on alternate days, and we randomly assessed breakfast, lunch, and dinner intakes for each resident. In Portugal, mid-morning snack, mid-afternoon snack and supper were also assessed. Meals of two complete days were recorded, considering one meat and one fish meal, at lunch and dinner. The double-weighing method emerged as the most suitable approach for assessing actual intake in NH compared to other methods such as consumption frequency questionnaires. This method provides a direct and reliable assessment of the true quantity of food ingested, as indicated by the review conducted by Ocké et al. [13]. The weighing also considered the individual components of each dish, such as the portions of meat, fish, or eggs; potatoes, rice or pasta; and vegetables. For mixed dishes, to consider the added fats and salt, the nutritional evaluation was conducted considering the quantities of ingredients per portion, as stated in the technical data sheets of the culinary preparations available in each care home. In the case of Spain, we used the food composition table made by Moreiras et al. [19], and for Portugal, we used the food composition table made by the Dr. Ricardo Jorge Institute (PortFIR INSA, Lisboa, Portugal) [20]. The parameters selected were total energy, total fat, saturated fatty acids (SFA), carbohydrates, total sugars (monosaccharides and disaccharides added to foods and beverages by the manufacturer, cook or consumer, and sugars naturally present in foods), protein and salt. The nutritional intakes of the individuals were compared to the reference values for people over 70, as elaborated for the calculation and adaptation to individual nutritional needs by the EFSA [14] and the WHO [15]. Individual energy requirements were calculated based on the equation proposed by the WHO [15] and the adequacy of energy intake was estimated based on the proportion of compliance with the energy requirements (i.e., adequate if the intake was 90–110% of requirements; inadequacy for intakes < 90% or >110% requirements).

### 2.4. Statistical Analysis

Categorical variables were described by their absolute (n) and relative (%) frequencies. The normality of continuous variables was assessed through the Kolmogorov–Smirnov test. Variables following a normal distribution were described by means, standard deviation, minimum and maximum; variables not following a normal distribution were described by median and respective interquartile range (25th percentile; 75th percentile). Comparisons of proportions were performed through the chi-square test and the comparison of means was done through the t-test, or the Mann–Whitney U test, according to the normality of the distribution of the variables. All statistical analyses were performed with IBM SPSS Statistics 26 software (SPSS, Inc., Chicago, IL, USA). A confidence interval of 95% and statistical significance defined by *p* < 0.05 were accepted.

## 3. Results

### 3.1. Sample Characteristics

Table 1 shows the characteristics of the 186 residents (118 women and 68 men). In the Spanish NH, the median age was 85.0 years (Percentile (P) 25; P75: 82; 88), as in Portugal (85.0 years (P25; P75: 80; 91). The oldest residents (4th quartile) were mainly women (70.0% in Spain and 90.4% in Portugal). In the Spanish NH, the most represented genders in the first and second quartiles were men (58.3% and 52.9%, respectively). In Portugal, the percentages of women were also higher in the first and second quartiles (73.3% and 77.2%, respectively). According to the BMI categories defined by Lipchitz (13), 32.8% had a normal weight and 8.1% were underweight. No significant differences were found between countries. In both countries, women were the most overweight group (59.4% in Spain and 83.8% in Portugal).

### 3.2. Nutritional Intake

Table 2 shows the means and medians of nutritional intakes for each region, calculated via the double-weighing method, and the percentage of older people who met the recommendations of the EFSA [14] and WHO [15].

#### 3.2.1. Energy Consumption

**Figure 1:**
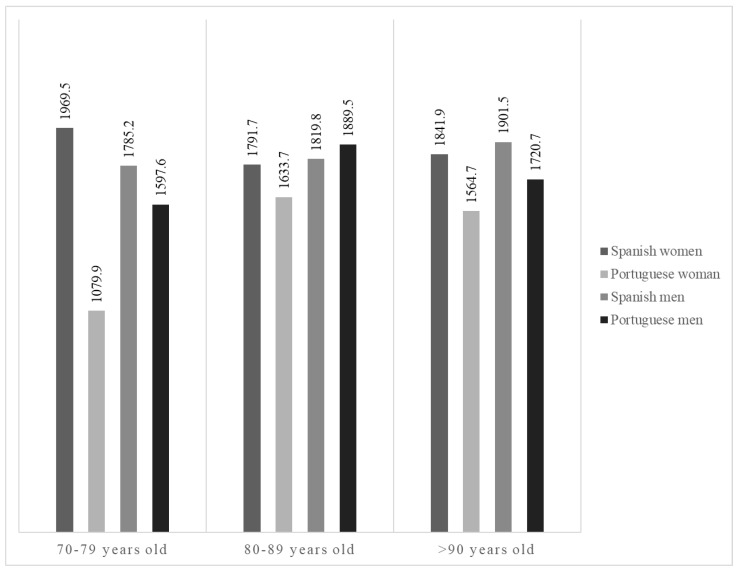
Energy intake (kcal/day) by age group.

The total energy intake was significantly higher in the Spanish care facilities (1842 kcal/day) than in the Portuguese facilities (1644 kcal/day) (*p* = 0.04). Intake was significantly lower (*p* = 0.030) in Portuguese women (1578 kcal/day, compared to 1760 kcal/day in Portuguese men). The intake of Portuguese women was also lower than that of Spanish women (1863 kcal/day) and Spanish men (1790 kcal/day). No significant gender differences were found in Spain. Figure 1 shows the intakes of Spanish and Portuguese women and men according to age. The highest calorie intakes correspond to the age groups of Spanish women aged 70 to 79 and Spanish men over 90. In general, Portuguese women had the lowest calorie intake, especially in the 70–79 age group. Less than 40% (38.9% of the Spanish residents and 34.3% of the Portuguese residents) had an energetic intake that could be considered satisfactory since it covered 100 ± 10% of their individual caloric needs.

**Figure 2:**
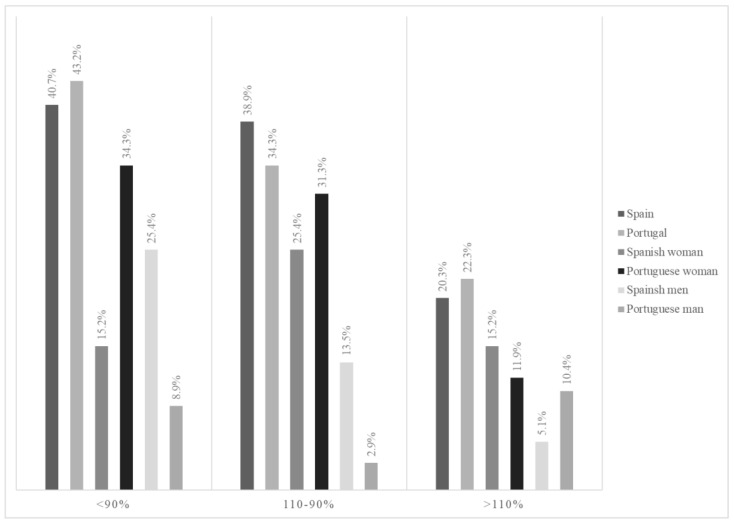
Distribution of residents according to the percentage of energy covered based on their individual calorie needs.

Figure 2 shows that most residents had an energy intake that covered less than 90.0% of their individual energetic requirements, with the largest group being Portuguese women. In contrast, 20.3% of Spanish residents and 22.3% of Portuguese residents had an intake of more than 10.0% of their individual energetic requirements; in this case, the group of Portuguese men stood out.

#### 3.2.2. Macronutrient Distribution

The percentage of energy derived from carbohydrates was higher in the Portuguese care facilities than in the Spanish facilities (60.5% vs. 56.0%), although as far as gender is concerned, there were no significant differences for country of residence. Carbohydrate intake was significantly higher at breakfast and lunch for Spanish residents than for Portuguese residents (66.7 g vs. 40.0 g (*p* < 0.001) and (104.6 g vs. 76.7 g *p* < 0.001), respectively. However, at dinner, intake was significantly higher for Portuguese residents (61.5 g vs. 32.7 g, *p* < 0.001).

Spanish residents consumed a significantly higher total fat proportion than Portuguese residents (24.0% vs. 20.4%, *p* < 0.001). In both regions, intake was higher in men (25.0% in Spain and 20.9% in Portugal) than in women (24.0% in Spain and 20.2% in Portugal). No significant gender differences were found.

**Figure 3:**
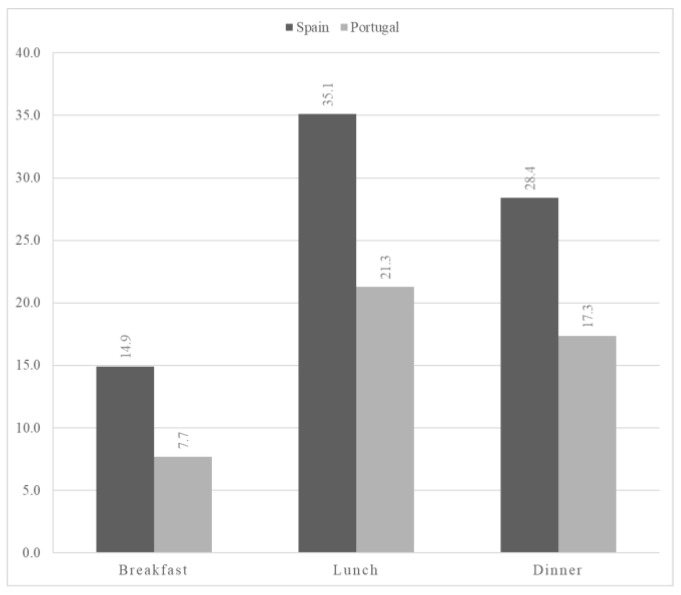
Distribution of protein intake per meal.

Daily protein intake (g/kg BW/day) was 1.05 in Spain and 0.9 in Portugal (*p* < 0.001). Women in Spanish care facilities had a significantly higher protein intake than Spanish men (1.1 g/kg BW/day vs. 0.9 g/kg BW/day) (*p* < 0.001). In Spanish NH, protein intake at lunch and dinner was within the 25.0–30.0 g of protein range, but not at breakfast (Figure 3). In Portugal, there were no significant gender differences regarding protein intake. It should be noted that, in Portuguese NH, many older people have morning and afternoon snacks, which may contain milk or dairy products and therefore also provide protein.

#### 3.2.3. Specific Nutrients

Total sugar intake was significantly higher (*p* = 0.009) for Portuguese than for Spanish residents (71.1 g/day vs. 59.9 g/day). With respect to gender, no differences were found among genders in each country, but Portuguese women consumed significantly more sugar than Spanish women (71.4 g/day vs. 59.6 g/day, *p* = 0.024).

Salt intake in the care facilities in Spain (5.8 g/day) was significantly higher (*p* = 0.001) than in Portugal (4.3 g/day). In both countries, there were no significant differences according to gender. At lunch there was a higher salt intake (2.9 g/day in Spain and 1.2 g/day in Portugal). There were no significant differences in SFA intake between the Spanish and Portuguese NH (*p* = 0.293).

### 3.3. Nutritional Adequacy

Table 2 shows the percentages of participants who complied with the nutritional recommendations. In Portugal, 54.5% of the residents have inadequate carbohydrate consumption, where the majority (52.9%) consumed more carbohydrates than was recommended. In Spain, the proportion of older adults with inadequate carbohydrate consumption was 33.1%, and the larger part of these (29.6%) had a carbohydrate intake above 60% of their total energy intake. Women showed the highest carbohydrate intake in both countries, but a high proportion of these were still within the recommended range (83.3% in Portugal and 57.7% in Spain).

In terms of total fat, the percentages of adequacy were similar between countries. In each subgroup, total fat intake was higher than 35.0% of total energy intake, the upper limit of the reference intake range (RIs). Intakes below 20.0% of total energy intake were observed at 46.2% and 44.9% for the residents in Portugal and Spain, respectively. Men showed a higher percentage of adequacy (54.2% in Spain and 52.1% in Portugal). In both countries, there was a higher percentage of women with an intake below the recommended range.

**Figure 4:**
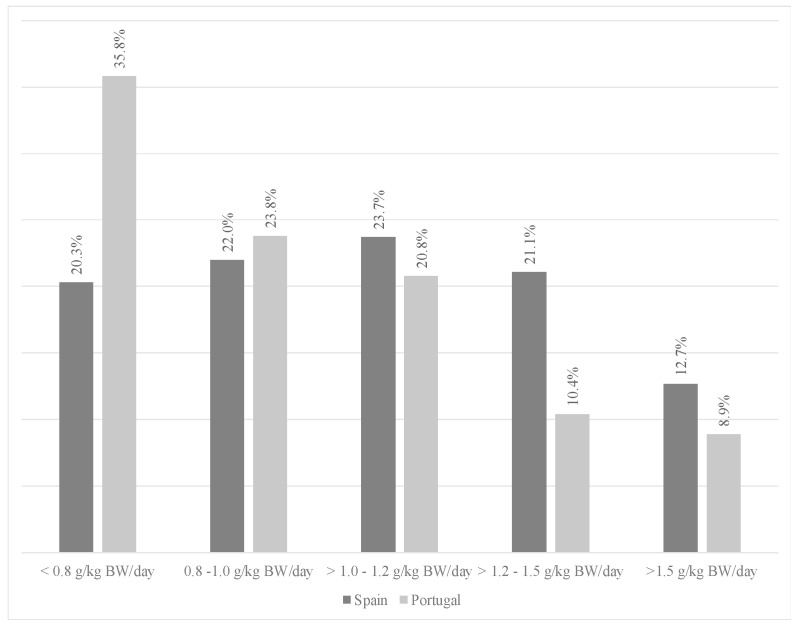
Distribution of residents by country in terms of protein intake (g/kg BW/day).

Salt intake was higher than was recommended (5 g/day) for 67.0% of residents in Spain and 32.3% of residents in Portugal, and women were found to have the highest salt intake. In both countries, more than 90.0% of residents showed adequate ranges of SFA. Figure 4 shows the distribution of residents in terms of protein intake (g/kg BW/day). Low proportions—20.3% and 35.8% of Spanish and Portuguese residents, respectively—had an intake below the EFSA recommendation [14] (0.83 g/kg BW/day) for healthy adults. Residents in Portugal mainly had an intake between 0.8 and 1.0 g/kg BW/day, while in Spain it was between 1.0 and 1.2 g/kg BW/day.

## 4. Discussion

Overall, it was observed that a high percentage of residents, both in Spain and Portugal, had inadequate energy intakes and macronutrient distributions. Moreover, Spain deviates from the EFSA recommendations [14] regarding salt intake, while surpassing the recommended protein intake (g/kg BW/day). It is important to note that exceeding the protein recommendation is not necessarily a negative aspect.

### 4.1. Energy Intake

Studies that have used the double-weighing method to assess intake in older people are scarce, although it is the ideal method to assess actual food intake [9]. In comparison with other studies that have followed this methodology, it was observed that the energy intake in the Portuguese care facilities was similar to or higher than those observed in Finnish, Belgian or Iberian facilities [21,22,23,24]. Energy intake in Spanish homes was higher than in Portugal and in the other countries mentioned above. In previous works in Spain [25,26,27], higher intake values were obtained than those observed in this study. Other studies analyzing energy intake by means of food frequency questionnaires or 24 h records showed lower intakes than those obtained in Poland [28] and Canada [29], and similar levels to those found in Turkey [30].

### 4.2. Individual Energy Requirements

It should be noted that 61.0% of Spanish residents and 65.5% of Portuguese residents do not have energy intakes adequate for their individual needs, even for those how are highly sedentary. This same trend was observed in other studies [21,23,29,31,32]. There is evidence of insufficient physical activity in care homes for older people [33]. Mirre den Ouden et al. [34] observed that residents spend most of their time sleeping, watching TV, or doing nothing. This reduction in physical activity will have a negative effect on institutionalized people and increase their dependency. To estimate their energy requirements, most European authorities determine the resting metabolic rate (RMR) using the Schofield formula, based on body mass and multiplying the RMR by a physical activity level (PAL) of 1.4, as in the present work [17,35]. This formula, like so many others, is designed for non-institutionalized people [17], and energy needs are not necessarily the same for people living in care facilities as they are for those living at home. Buckinx et al. [32] calculated the caloric needs of NH residents via indirect calorimetry and estimated a PAL of 1.29 ± 0.3—a value lower than that found in healthy independent people [36]. In sick older people, the PAL was as high as 1.23 [36]. This value is slightly lower than the minimum value necessary for basal physiological functions, set at 1.27 by the WHO [15]. In the ESPEN guide [37], 30 kcal/kg BW/day intake is indicated for older people; however, in the review by Gaillard et al. [36], a decrease in energy requirements is described, and it was deemed more appropriate to place it at kcal/kg BW/day. This decrease is influenced by having less fat free mass (FFM), more fat mass (FM) and less PAL [36]. There is no clear consensus when it comes to accurately estimating the individual energetic needs of older people due to the high heterogeneity of this population. As might be expected, lower REE and lower daily physical activity mean that the energy needs of institutionalized people are also lower. Taking these factors into account, we adjusted the PAL of the residents based on the methods of the previously described studies using a PAL of 1.27 instead of 1.4, showing that the number of NH residents covering <90% of their individual energy needs decreases by 10.0–15.0%, while the number of residents with an intake above 110% of their individual caloric needs increases by 15.0–20.0%. In the case of Portuguese women aged 70–79, with the lowest energetic intake, this probably reflects a very small sample, and thus cannot be generalized. Moreover, in Portugal, most women are only institutionalized when they can no longer take care of themselves due to health limitations, unlike widowers, for example, who may choose to go to an NH of their own choice. In other words, this female subgroup is more prone to morbidity, associated with anorexia. In addition, as they are more accustomed to cook more often, they are likely to be more demanding about the meals served, and end up wasting more; thus, as we have detected in our sample, they end up having insufficient intake.

In the calculation of energy intake, it was not possible to consider the intake of food consumed by residents outside the homes, or food offered by visiting relatives and friends. The food eaten in addition to that offered by the institutions, together with the inactivity of the residents, may also result in the prevalence of obese and overweight residents.

### 4.3. Macronutrient Distribution

#### 4.3.1. Protein Intake

The distribution of macronutrients is essential for older people to maintain optimal health. Protein is the most important macronutrient in older adults. There are not many intake data available for institutionalized NH residents, even though this group is at risk of low protein intake [38]. The amount of protein required for people over 65 is under discussion by scientific societies [14,15] and by experts [38]. The recommended protein intake ranges from 0.83 to 1.2 g/kg BW/day, as per EFSA guidelines [14]. Notably, 77.0% of Spanish residents and 60.0% of Portuguese residents surpass the recommended threshold of 0.83 g/kg BW/day, aligning with EFSA recommendations [14]. Based on the recommendations of PROTE-AGE, [38] the most adequate intake is between 1.0 and 1.2 g/kg BW/day, which was met by 23.7% of the Spanish sample and 20.8% of the Portuguese sample (Figure 4), meaning that more than 70.0% of residents did not meet these recommendations [38]. Another study carried out in Spain (Granada, South Spain) [39], which assessed protein intake in NH residents, showed a protein intake of 0.92 g/kg BW/day for men (62.5 g/day) and 1.0 g/kg BW/day for women (57.5 g/day). These results are similar to those found in this study for men (0.97 and 0.98 g protein/kg BW in Spanish and Portuguese residents, respectively) and Spanish women (1.1 g protein/kg BW), but higher than those found in Portuguese women (0.88 g protein/kg BW). In contrast, total protein intake per day was higher in Spain and Portugal as in other studies [21,22,25,40]. In another study from the Mediterranean area of Spain [41], carried out with adults over 65 years of age, NH residents’ mean protein intake was 71.4 g/day. Thus, it was lower than that assessed in the Spanish NH residents in this study (82.0 g/day), but higher than that observed in the Portuguese nursing home residents (61.0 g/day). However, in several studies [29,32], intakes ranging from 83.1 g/day to 99.6 g/day were reported, which are higher than in the present study. Authors such as Loenneke et al. and Moore et al. suggest that an adequate intake is between 25.0 and 30.0 g of protein at each main meal [42,43]. This recommendation for protein intake was not met in the present study on Portuguese nursing homes, as was the case in the work of Rodriguez et al. [39].

#### 4.3.2. Total Fat Intake

Total fat intake in residents of Spain and Portugal was lower than that found in other studies in the Iberian Peninsula, Turkey, and Poland [21,23,29,31]. The impact of a low-fat diet in older people is not sufficiently studied, as the focus of research tends to be on excess intake and its metabolic or cognitive implications [44]. Participants in this study had a lipid intake close to the lower limit of the EFSA’s recommended range [14]. As per EFSA considerations, no nutritional deficiencies are observed when fat intake is around 20.0% of total energy, although the risk of a mineral or fat-soluble vitamin deficiency is high [14]. In older people, the consequences of a diet low in fat have been linked to poorer hearing function and a higher prevalence of neurodegenerative diseases, depression, bipolar disorders, schizophrenia, or Alzheimer’s due to the lower absorption of vitamin A, E and PUFAs (polyunsaturated fatty acids) [45]. The trend to reduce fat intake to prevent the risk of cardiovascular disease (CVD) led NHs to prioritize the inclusion of low-fat products, such as skimmed dairy and dairy products, on menus, thus leaving oils, mainly olive oil, as the main source of fat in the diet. The use of skimmed dairy products is less and less justified in older people. According to the researchers of the PREDIMED study [46], the inclusion of the consumption of whole dairy within the recommendations may have a protective role against the development of metabolic syndrome, as no negative associations with abdominal obesity, dyslipidemia, hypertension, or factors related to increased cardiovascular disease have been found. This evidence suggests that it is not so much the quantity of lipids that needs to be monitored as their quality in order to achieve a healthier diet, specifically as regards saturated fatty acids (SFA). Most dietary guidelines recommend that SFA intake should be less than 10.0% of total energy [15]. However, the EFSA suggests that their intake should be as low as possible [45]. In participants from both countries, this requirement was met. The values recorded were lower than those observed in other care facilities [26,31,32,39,41]. The amounts of SFA ingested were adequate, but its low presence is also a consequence of the low intake of total fat.

#### 4.3.3. Carbohydrate Intake

In relation to carbohydrates, the EFSA’s scientific position states that intake should be higher than 100 g/day to avoid the onset of ketosis. The range of intake should be between 45 and 60% of total energy, a value compatible with normal glucose levels in healthy subjects or those with metabolic syndrome [14,47]. Within the examined care facilities, the findings indicate that Spanish participants demonstrated a higher total carbohydrate intake, whereas Portuguese participants exhibited greater sugar intake. This highlights a nuanced distinction, emphasizing that the observed differences in carbohydrate intake are specific to total carbohydrates, and do not necessarily extend to sugar consumption. In the care facilities of both countries, a higher intake was detected in comparison with similar studies [22,26,29,32,39,41]. It is very important to monitor the quality of the carbohydrates offered. According to a recent meta-analysis, the inclusion of two to three servings of wholegrain cereals per day was associated with a 21.0% reduction in the risk of cardiovascular events [48]. The idea is that using an intervention based on nutrition education to help empower residents to include higher quality cereals may facilitate an increase in the intake of whole grain cereals. Interventions of this sort may improve the nutrition of older NH residents in the future.

In this research, we assessed the intake of total sugar. Based on the recent statement on the EFSA’s scientific position, intakes of total sugars by the core group (fruit, vegetables, cereals and derived products, and dairy and dairy products) have not been associated with an increased risk of hypertension, CVD, gout, dyslipidemia, obesity, type 2 diabetes or nonalcoholic fatty liver disease/nonalcoholic steatohepatitis [49]. Not many studies have assessed total sugar intake in older people. The most representative one was conducted by Goletzke et al., who followed a cohort of older people for 5 years without finding any association between total sugar intake and changes in triglycerides or high-density lipoprotein cholesterol [50]. The latest evidence indicates that the intake of less than 90 g of total sugars is adequate for adults [51]. Participants in this study were found to meet these recommendations. In the ANIBES study, it was found that 85% of the total sugars ingested came mainly from dairy and dairy products, fruit, sugars and sweets, cereals and cereal products, and vegetables [52]. In the present study, the intake of fruit and vegetables was found to be low. The low intake of total sugars might have been influenced by the low intake of fruit and vegetables observed during the fieldwork. Regardless of the intake of total sugars, the amount of free or added sugars should be as low as possible. Most breakfasts in Spanish nursing homes included ultra-processed foods such as biscuits; indeed, an average weight of 75 g of biscuits was offered at breakfast, which is not advisable for the reasons mentioned above.

#### 4.3.4. Specific Nutrients

Regarding salt intake, EFSA indicates that the safe intake for the adult population should not exceed 5 g salt/day [53]. However, there are no specific recommendations for older people. With age, renal and cardiovascular function declines that may make blood pressure more sensitive to increases in salt intake [54]. In this research, 66.7% of Portuguese residents consumed less than 5 g salt/day. In Spain, the intake of 67.0% of the residents was higher than recommended, which implies a risk of cardiovascular disease [54]. These data are even more relevant if we consider that only the intake of salt present in the food included in the NH menus was recorded; hence, the intake of Portuguese and Spanish residents might be higher. In comparison with studies conducted in other NH, salt intake was lower than that reported in Canada (10.8 g/day) [30] and Spain (6.6 g/day) [39], and slightly higher than in nursing homes in Poland (5.5 g/day) [29] or in another study in Spain (3.0 g/day) [25]. It is important to clarify that the study was based on the technical data sheets of the dishes, but these may not be strictly adhered to by the cooks. The most rigorous method of analyzing sodium intake would be through urinary measurements. Moreover, in Portugal, some older adults have their own saltshakers and add salt on the table. Intakes have been found to be higher than the EFSA recommendation, and therefore great attention should be paid to the food consumed by NH residents during their daily activities outside the care facility, as their total intakes might be even higher.

Notwithstanding the valuable data presented in this paper, it would be interesting to analyze the adequacy of micronutrients, too, but to do this would require that information be collected for a longer period of time, given the intra-individual variability that exists in the consumption of such nutrients. Moreover, we acknowledge that we excluded mixed dishes because it was impossible to separate the quantities of meat/fish, potatoes/rice, or pasta and vegetables. It is conceivable that these meals may have varying proportions of different components.

## 5. Conclusions

After assessing the intakes of NH residents in Spain and Portugal, it was found that these intakes need to be adjusted to meet energy and nutrient recommendations. A high percentage of residents ingest less energy and fat than recommended, and exceed the recommended intake of carbohydrates and salt. Interestingly, protein intake (g/kg BW/day) exceeds the recommended values. Considering these findings, it becomes imperative to refine menu planning by aligning energy intake with the actual expenditure of nursing home residents. This strategic adjustment aims to enhance the provision of foods rich in healthy fats. Furthermore, the increased scrutiny and quality control of the menus offered is recommended for optimal nutritional care.

## Figures and Tables

**Table 1:** Characterization of study participants.

Participants (*n* = 186) *	Spain	Portugal
Age (years), median	85.0	85.0
Percentile 25	82.0	80.0
75	88.0	91.0
Male, *n* (%)	52 (44.1)	16 (23.5)
Female, *n* (%)	66 (55.9)	52 (76.5)
Weight (kg), median	75.0	66.0
Percentile 25	65.9	57.0
75	82.0	77.7
Male median	76.2	77.2
Female median	72.0	64.0
BMI (kg/m^2^), median	28.6	27.3
Percentile 25	24.5	24.3
75	31.7	30.6
Underweight (BMI < 22), *n* (%)	8 (6.7)	7 (10.2)
Normal (BMI 22–27), *n* (%)	36 (30.5)	25 (36.7)
Overweight (BMI > 27), *n* (%)	74 (62.7)	36 (52.9)

* There were no statistically significant differences between Spanish and Portuguese participants; BMI—body mass index.

**Table 2:** Recommended daily intake (RDI), actual daily intake and percentage adequacy among study participants.

	Spain										Portugal		
						Percentage of Residents within Adequacy					Percentage of Residents within Adequacy		
Nutrient	RDI	Mean	SD	Median	IQR	%	95% CI	Mean	SD	Median	IQR	%	95% CI
Energy (kcal)				1842 *	1575–2056					1644	1367–1890		
Carbohydrates (% of energy)	45–60 ^a^	56.0	0.71	56.0 ***	50.7–62.0	66.9	58.3–75.5	59.3	0.6	60.5	56.8–63.1	45.5	33.4–57.7
Carbohydrates (g)	>100	258.1	6.1	250.7	209.5–300.2			243.1	7.2	239.6	199.6–274.6		
Total sugar (g)	<90	66.0	2.1	59.6	50.6–75.6	88.1	82.2–94.0	72.3	2.4	71.1	56.4–85.3	86.7	78.5–95.0
Total fat (% of energy)	20–35 ^a^	24.1	0.7	24.0 *	17.0–31.0	54.2	45.2–63.3	20.7	0.5	20.4	17.8–23.3	53.8	46.5–61.0
Total fat (g)		48.7	1.7	45.0 ***	34.6–62.6			38.3	1.5	39.3	29.0–46.2		
SFA (% of energy)	ALAP ^b^	6.9	0.2	7.2	4.8–8.6			7.49	0.2	7.3	6.3–8.2		
Protein (% of energy)	10–15	17.8	0.2	18.0 ***	16.0–20.0	17.8	10.7–24.8	14.8	0.3	14.2	12.8–16.2	54.4	42.2–66.5
Protein (g)		82.2	2.3	77.3 ***	67.2–92.9			61.0	2.1	62.5	48.3–73.8		
Salt (g)	5 ^c^			5.8 ***	4.6–6.7	33.0	24.4–41.6			4.3	3.8–5.3	67.7	56.2–79.0

* Note: The prevalence of adequacy is expressed in percentage and the 95% confidence interval (CI). ^a^ RIs: Reference intake range for total fat and carbohydrates (EFSA). ^b^ ALAP: as low as possible (EFSA). ^c^ AI: adequate intakes (EFSA). * *p* < 0.05; *** *p* < 0.001.

## Data Availability

The raw data supporting the conclusions of this article will be made available by the authors, without undue reservation.
